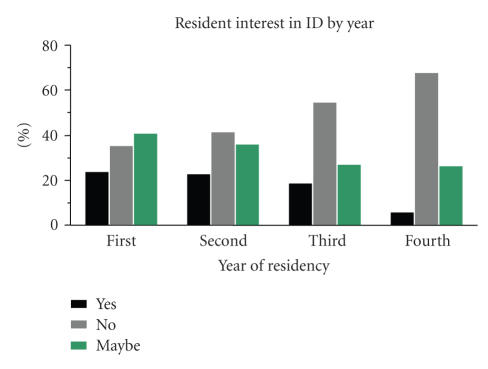# Awareness of Infectious Diseases in Obstetrics and Gynecology Among Residents and Residency Directors

**DOI:** 10.1155/IDOG/2006/42967

**Published:** 2006-11-19

**Authors:** Richard H. Beigi, Galen E. Switzer, Larraine Presley, David E. Soper

**Affiliations:** ^1^Department of Obstetrics, Gynecology, and Reproductive Sciences, Magee-Womens Hospital of the University of Pittsburgh Medical Center, University of Pittsburgh School of Medicine, Pittsburgh, PA 15213, USA; ^2^Department of Medicine, University of Pittsburgh School of Medicine, Pittsburgh, PA 15213, USA; ^3^Veteran's Affairs Medical Center, University Drive C, Pittsburgh, PA 15240, USA; ^4^Department of Obstetrics and Gynecology, MetroHealth Medical Center, Case Western Reserve University, Cleveland, OH 44109-1998, USA; ^5^Department of Obstetrics and Gynecology, Medical University of South Carolina, Charleston, SC 29425, USA

## Abstract

Awareness of the subspecialty of infectious diseases in obstetrics and gynecology is low among United States residents and residency directors. *Objective*. Given the burden of infectious diseases on women's health, we sought to assess current awareness, interest, and perceived value of the subspecialty of infectious diseases in obstetrics and gynecology among current United States obstetrics and gynecology residents and residency directors. *Methods*. Two separate surveys addressing awareness, perceived value and interest in the subspecialty were sent to (1) a random 20% sample of obstetrics and gynecology residents and (2) all obstetrics and gynecology residency directors. *Results*. Seventy percent of the residency directors were familiar with the subspecialty and 67.0% placed value on infectious disease specialists in an academic department. Thirty percent of the residents reported awareness of the subspecialty. Thirty-six percent of residency directors reported that medical infectious disease specialists deliver formal education to the obstetrics and gynecology residents. *Conclusion*. United States obstetrics and gynecology residents and residency directors have a low awareness of the subspecialty. An open niche exists for formal education of residents in infectious diseases in obstetrics and gynecology by department specialists. These findings can be incorporated into ongoing recruitment efforts for the subspecialty of infectious diseases in obstetrics and gynecology.

## INTRODUCTION

Infectious diseases have been and are currently responsible for a large percentage of common obstetrical and gynecological morbidity and occasional mortality [1]. Moreover, a sizable percentage of total health-care dollars are spent on women's health targets (either directly or indirectly) and infectious diseases control and management [1–5]. Recent conservative estimates of costs of infectious diseases in women are in excess of a billion dollars each year [6].

As a current affiliate of the American College of Obstetricians and Gynecologists (ACOG), the Infectious Diseases Society for Obstetrics and Gynecology (IDSOG) was created in 1973 with the purpose of bringing together professionals in the field of obstetrics and gynecology that are interested and have training in the study and practice of infectious diseases in women. Activities of the society include the scientific study of the field, promulgation of knowledge regarding the area of infectious diseases in women, and the facilitation of relationships between clinicians/investigators focused on this area of expertise. The society currently has 122 active members, and ongoing recruitment is a priority.

We sought to gather data regarding current awareness, interest, and perceived value of the subspecialty of infectious diseases in obstetrics and gynecology (Ob/Gyn-ID) among current United States residency directors and residents. Residency directors were included given their vital role as resident advisors for career planning and their primary responsibility as resident education coordinators. The findings are being used to fulfill two main goals: (1) to foster ongoing recruitment efforts for the subspecialty of Ob/Gyn-ID by the IDSOG and (2) to assess the perceived value of the subspecialty given the large burden of infectious diseases in women.

## MATERIALS AND METHODS

Two focused surveys were constructed addressing the two descriptive goals of the study described previously. A random sample of 20% of the current United States resident pool was generated in partnership with ACOG. Questions addressing current resident year, gender, geographic location, plans for practice including plans for fellowship training, awareness in the subspecialty of Ob/Gyn-ID, interest in nontraditional fellowships (non-maternal-fetal medicine (MFM), reproductive endocrinology and infertility (REI), gynecologic oncology (GYN-ONC), and urogynecology (URO-GYN)), and potential interest in this subspecialty were included. All current United States obstetrics and gynecology residency directors received a separate survey. The residency directors' questions included years in the position, awareness, and perceived value of Ob/Gyn-ID, recollection of resident interest in opportunities for fellowship training in infectious diseases and their knowledge of how to direct those interested residents.

Both surveys were field-tested for comprehension, content, and applicability and noted to be acceptable at the institution of the first author. One thousand resident surveys and 256 residency director surveys were mailed in January of 2005. The last survey response was collected in June, 2005.

Sample size for the resident pool was estimated given consideration of what would constitute a representative sample for a survey whose goals are primarily descriptive. Collation and analysis of data was performed using StatView (version 5.0.1, SAS Institute Inc, Cary, NC, USA). Summary statistics were used for the description of data, and analysis of the data was performed using *χ* ^2^ testing for differences in proportions and simple linear regression for trend analysis.

## RESULTS

**Figure 1:**
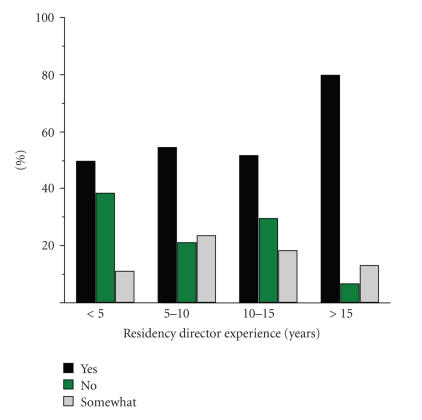


One hundred and sixty-four of the 256 (64.0%) residency directors completed and returned the survey. Nearly half (49%) of the residency director respondents had been in the position for less than 5 years, another quarter (26%) had served in that role for 5–10 years, and the remaining 25% for more than 10 years. Overall, 70% of the respondents were at least somewhat familiar with the specialty (Figure 1). In terms of perceived value of the subspecialty, 67% of the respondents stated that having a specialist in their department with specific training in infectious diseases was valuable. Eight percent reported that residents had asked them about training opportunities in infectious diseases, and 62% were aware of how to guide the residents.

**Figure 2:**
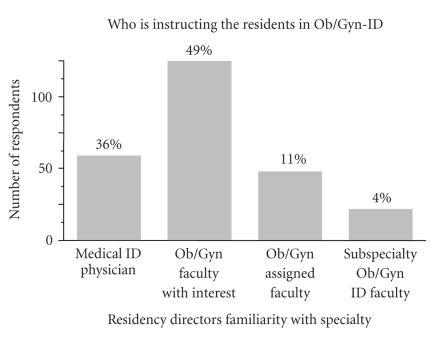


Formal education of obstetrics and gynecology residents on infectious diseases issues is an area of large potential impact for clinical competence. Therefore, we assessed who is primarily educating the current residents on these topics. Figure 2 shows the results of this query. Of note, 36% of residency directors reported formal education on obstetrical and gynecological infections was given by medical infectious diseases physicians.

Of the 1000 residents targeted, 354 (35.4%) completed and returned the survey. Nine were returned due to an incorrect address, making the overall return rate 36.0%. All geographic regions of the United States were represented, with the majority of the responses coming from the Northeast (32%) and Midwest (24%). The average age of the respondents was 30, with a standard deviation of 3.3 and a range of age 22–47. Seventy-four percent of the respondents were female and 26% were male.

The majority of respondents were first, second, and third years residents (90%). Fifty-one percent were planning on private practice settings, 21% were planning on an academic career, and 28% were undecided. One hundred thirty-four (38%) of respondents planned on pursuing fellowship training and 81 (23%) were currently undecided. Of those planning on pursuing fellowship training, approximately 13 (10%) planned on doing “nontraditional” fellowships (non-MFM, REI, GYN-ONC, URO-GYN) including family planning and minimally invasive surgery. Two-hundred forty-eight of the 354 respondents (70%) were completely unaware of the subspecialty of Ob/Gyn-ID, and 42 (12%) reported hearing about it but not being completely familiar with the subspecialty. Of the 30% that were at least somewhat familiar with the subspecialty, 10 (33%) had considered Ob/Gyn-ID as an option. Greater than 50% of the total resident sample indicated that if fellowship training options were available in Ob/Gyn-ID they may have potential interest. Notably, the percentage of respondents interested in this fellowship training significantly decreased throughout the residency training period (*P* = .01) (Figure 3).

## DISCUSSION

The findings of these surveys demonstrate that current US obstetrics and gynecology residents have a low awareness of the subspecialty of Ob/Gyn-ID. Awareness of the subspecialty of Ob/Gyn-ID among current US obstetrics and gynecology residency directors is relatively low (70%). Perceived value among residency directors of the specialty was also relatively low (67%). When asked about training opportunities in infectious diseases, only 62% of residency directors were aware of how to guide the residents. These surveys highlight significant deficiencies in awareness and perceived value of Ob/Gyn-ID among residents and residency directors and are undoubtedly in contrast to awareness, perceived value, and knowledge about training opportunities for the traditional fellowships (MFM, REI, GYN-ONC, URO-GYN), which is likely 100%.

Of the resident responders, more than half stated they may have potential interest in the subspecialty if fellowship training options were available. The resident interest was seen predominantly in the early years of residency training (first and second years) and waned considerably in the later years. There are potential reasons for this finding. Early in-training residents may have ideas of what interests them and after experience realize more precisely their interests. This is in line with the recent findings of Gilpin addressing resident attrition in that the majority of Ob/Gyn residents that changed specialties did so in the first two years of training [7]. Cain et al also found waning interest in academic careers among Ob/Gyn residents as residency progressed [8]. Financial considerations such as the high prevalence of large resident debt from educational loans combined with decreasing reimbursements and compensation may also contribute. These findings highlight the need to begin recruitment efforts in the early years of residency training to engage interested residents to potentially improve recruitment.

Nearly 40% of current United States obstetrics and gynecology residents that responded planned on pursuing fellowship training, with the majority seeking fellowship positions in the “traditional” fellowships (MFM, REI, GYN-ONC, URO-GYN). This is not surprising as their obvious applicability was noted through role-modeling during residency. Reports from the late 1990's noted decreasing rates of fellowship interest and matriculation in MFM and REI and increases in GYN-ONC [9]. Our data are potentially biased given the low-resident response rate and may represent a sample of more academically focused residents given the content of the survey. These data may also represent a portion of the resident pool that was interested in unique training opportunities. The reported interest in fellowship training overall is encouraging given the improvements noted in education, specialty development, and academic contribution with the formation of subspecialties in obstetrics and gynecology [10]. It has been recognized for years that obstetrician-gynecologists are underrepresented in academic medicine and medical research [11]. Given this, fellowships in academically geared specialties such as infectious diseases remain paramount to the continued advancement of obstetrics and gynecology.

The residency director survey highlighted an open niche in terms of formal education of residents on common infectious diseases topics. This conclusion is based on the fact that 36% of residency directors reported that formal infectious diseases education was given by cross-discipline specialists (medical infectious diseases physicians). This is not to suggest that medical infectious diseases specialists are not knowledgeable or able to teach effectively. However, Ob/Gyn-ID subspecialists have unique knowledge, training, and experience about pregnancy and gynecologic and postoperative infections and are therefore more likely to impart relevant and focused information for trainees.

Several limitations to the current investigation are worth notice. Data generated by survey research are always limited by the return rate and the inherent bias in selective response. The return rate among residency directors (64%) is relatively high, compares favorably with other publications of residency director surveys, and provides reliable and useful insight [12]. The resident return rate is substantially lower and does question the validity of the findings. As stated earlier, this low return rate may represent an academically focused subset of residents, biasing our results towards those resident attitudes. We, unfortunately, were unable to quantify this potential bias due to the random nature of this sample and the inability to effectively compare the nonresponders to the responders. With these limitations in mind, the return rate approximates other resident survey return rates in the literature [13]. Moreover, the resident return distribution corresponds to the higher density of residents in the Northeast and Midwest, suggesting that the data represent a geographically random sample [14]. Regardless, the low level of awareness among this potentially academically focused sample provides insight and may suggest even lower awareness of Ob/Gyn-ID among the entire resident sample.

Awareness of the subspecialty of Ob/Gyn-ID among current United States obstetrics and gynecology residency directors and residents was low. The reported potential resident interest in Ob/Gyn-ID fellowship training is mainly concentrated in the early years of training. In addition, an open niche exists for resident education by Ob/Gyn-ID subspecialists on common and complicated infectious diseases in obstetrics and gynecology. These surveys identify areas in which the IDSOG should focus to improve visibility and recruitment for this subspecialty.

## Figures and Tables

**Figure 3:**